# Association between depression and sleep quality in patients with systemic lupus erythematosus: a systematic review and meta-analysis

**DOI:** 10.1007/s11325-021-02405-0

**Published:** 2021-05-25

**Authors:** Rulan Yin, Lin Li, Lan Xu, Wenjie Sui, Mei’e Niu, Rong Xu, Chomphoonut Srirat

**Affiliations:** 1 Department of Rheumatology, The First Affiliated Hospital of Soochow University, 899th Pinghai Road, Suzhou, 215006 China; 2 Faculty of Nursing, Chiang Mai University, 110/406 Inthavaroros Road, Suthep, Muang District, Chiangmai, 50200 Thailand; 3 School of Nursing, Taizhou Polytechnic College, Taizhou, China; 4 Department of Nursing, The First Affiliated Hospital of Soochow University, Suzhou, China

**Keywords:** Depression, Sleep quality, Systemic lupus erythematosus, Meta-analysis

## Abstract

**Background:**

Currently, there is no consistent understanding of the relationship between depression and sleep quality in patients with systemic lupus erythematosus (SLE). This study aimed to explore the correlation between depression and sleep quality in SLE patients.

**Methods:**

Five English (PubMed, Web of Science, EMBASE, Cochrane Library, and CINAHL) databases were systematically searched from inception to January 12, 2021. Two authors independently screened publications and extracted data according to set inclusion and exclusion criteria. Statistical analyses were performed with STATA 16.0. Data were pooled using a random-effects model.

**Results:**

A total of 9 identified studies matched the inclusion criteria, reporting on 514 patients with SLE in the analysis. A moderate correlation of depression with sleep quality was found (pooled *r* = 0.580 [0.473, 0.670]). Compared to good sleepers, patients with SLE and poor sleep quality had higher levels of depression (standardized mean difference =  − 1.28 [− 1.87, − 0.69]). Depression was associated with subjective sleep quality (*r* = 0.332 [0.009, 0.592]), sleep latency (*r* = 0.412 [0.101, 0.649]), sleep disturbances (*r* = 0.405 [0.094, 0.645]), daytime dysfunction (r = 0.503 [0.214, 0.711]), the four dimensions of Pittsburgh Sleep Quality Index (PSQI), while no significant correlation was found in the other three PSQI dimensions.

**Conclusion:**

Depression had a moderate correlation with sleep quality in patients with SLE. Patients with poor sleep quality tended to have higher level of depression than that of good sleepers. Awareness of the correlation may help rheumatology physicians and nurses to assess and prevent depression and improve sleep quality in patients with SLE.

## Introduction

Systemic lupus erythematosus (SLE), the most common form of lupus, is a chronic, multi-organ, systemic autoimmune disease with unknown etiology and heterogeneous clinical presentation, affecting almost all tissues and organ systems, occurring predominantly in young adult women, with a female-male ratio of more than 9:1 [1, 2]. It was found in a 2020 meta-analysis that sleep quality of SLE patients is worse than that of the general population [3]; 56.0–80.5% of patients with SLE reported sleep disturbances and poor sleep quality [4]. Previous studies reported that sleep disturbances may lead to the aggravation of cardiovascular disease in patients with SLE [5]. Lack of sleep indicates a deterioration of disease activity [6]. Therefore, it is of great significance to identify the causes of poor sleep in patients with SLE so that targeted interventions can be taken to improve their sleep quality. However, the causes of sleep disorders have not been completely identified [7]. Meanwhile, psychological/social factors, particular depression, have been observed as the most frequent possible causes of sleep disorders in SLE [8–11]. As one of the most frequently observed neuropsychiatric disorders [12], depression affects 35.0% of patients with SLE [13]. In addition, SLE patients have more depressive symptoms than healthy women [14]. Cervilla et al. [14] reported that worse subjective quality of sleep was associated with more depressive symptoms in SLE. Wu et al. [3] also displayed that depressed patients with SLE have poorer sleep quality than that of the general population. However, Moraleda et al. [15] did not find a significant correlation with depression and sleep quality in patients with SLE. Given the importance of a clear association between depression and sleep quality in patients with SLE, it is necessary to conduct a synthesis on the empirical studies addressing this topic.

The Pittsburgh Sleep Quality Index (PSQI) [16], which consists of 19 items, is the most widely used instrument to evaluate sleep quality in clinical populations. The PSQI assesses seven dimensions: subjective sleep quality, sleep latency, sleep duration, habitual sleep efficiency, use of sleeping medication, sleep disturbances, and daytime dysfunction. Its clinemetric and clinical properties suggest utility both in psychiatric clinical practice and research activities, and has been widely applied to assess sleep quality in patients with SLE. However, despite having been measured in many studies, the degree of sleep quality in each domain has differed among different samples [3]. To the best of our knowledge, there is no comprehensive review on the relationship between depression and sleep quality as assessed by the PSQI in patients with SLE. Thus, with accumulating evidence, we performed a systematic review and meta-analysis to answer the following two questions: (i) Is there an association between depression and sleep quality in patients with SLE and (ii) Is the depression level higher in groups with poor sleep quality compared to good sleep quality.

## Methods

The systematic review and meta-analysis was conducted in accordance with the recommendations of the Preferred Reporting Items for Systemic Review and Meta-Analyses (PRISMA) guidelines [17].

### Search strategy

Up to January 12, 2021, five English databases were searched, including PubMed, Web of Science, EMBASE, Cochrane Library, and CINAHL. The search terms were combinations of the following: sleep (sleep problems or sleep disturbances or sleep quality or sleep efficiency or sleep latency or sleep difficulties), depression (depression or major depression disorder or depressive symptoms or mood disorder), and SLE (lupus or SLE or systemic lupus erythematosus). The reference lists of included studies were also reviewed for additional studies.

### Inclusion and exclusion criteria

Original studies were included if they (1) were cross-sectional or case–control or longitudinal studies (only the data at baseline were extracted) on patients with SLE patients; (2) reported the association between depression and sleep quality with the Pearson or Spearman correlation coefficient (*r*), or mean ± standard deviation (SD) of depression on both good sleep quality group and poor sleep quality group; (3) used PSQI to evaluate sleep quality; (4) used a validated scale to measure depression; (5) were published in the English language.

Exclusion criteria were the following: (1) studies were meta-analyses, reviews, case reports, qualitative studies, comments, editorial letters, or conference abstracts; (2) studies did not meet the inclusion criteria; (3) studies reported unusable or duplicate data. When there were different studies in the same unit and the same sample, the most recent was selected.

### Data extraction and quality assessment

According to the titles and abstracts, two authors independently decided whether to include articles by reading the abstract and further full-text examination, and independently extracted the following information from each article: first author, publication year, country, study design, aim, diagnostic criteria of SLE, source of the patient, sample size and percentage of female participants, age, disease duration, disease activity, corticosteroids use, cumulative disease damage, measurement, cut-point, prevalence and score of depression, cut-point and score of PSQI, prevalence of poor sleep quality, Pearson’s *r* or Spearman’s correlation coefficient (*r*) between depression and sleep quality, depression score both in good and poor sleep quality, and the number of patients in each group (good and poor sleep quality). Quality assessment was conducted alongside data extraction using a modified version of the Newcastle–Ottawa Scale (M-NOS) [18]. Studies were judged to be at low risk of bias (≥ 3 points) or high risk of bias (< 3 points). Any disagreements in data extraction and quality assessment were resolved through discussion between the two reviewers or adjudication with a third reviewer.

### Outcome measures

The outcomes were depression assessed with validated assessment tools (e.g., Hospital Anxiety and Depression Scale-Depression [HADS-D], the nine-item Patient Health Questionnaire [PHQ-9], Center for Epidemiological Studies Depression scale [CES-D], Hamilton rating scale for depression [HAM-D]), and sleep quality assessed by PSQI.

### Statistical analysis

Meta-analysis was performed with STATA 16. As random-effects model was preferable and could provide wider confidence interval (CI) [19], this model was conducted to pool *r* and continuous outcomes in this study. For correlation coefficients, Pearson correlation coefficients and Spearman correlation coefficients were all transformed into Pearson correlation coefficients. Pooled estimates of the Pearson coefficients were calculated by Fisher’s exact test *r*-to-*z* transformation [20]. All of the values were weighted with the inverse of the variance of the correlation coefficients, after which, the pooled *r* of the overall value were transformed back for presentation. For the continuous outcomes, we used standardized mean difference (SMD) with 95% CIs. *I*^2^ was used to assess between-study heterogeneity, with thresholds of 25% (low heterogeneity), 50% (moderate heterogeneity), and 75% (high heterogeneity) [21]. As the seven dimensions of PSQI indicated different aspects of sleep quality, this study separately summarized and analyzed 7 domains. Sensitivity analysis and subgroup analysis were performed to find the source of heterogeneity. Funnel plots and the Egger’s test were combined to evaluate for publication bias if the number of included studies is greater than or equal to 10 [22, 23], as the power of these tests is too low to distinguish chance from real asymmetry if less than 10 studies are included [24].

## Results

### Study selection

**Fig. 1:**
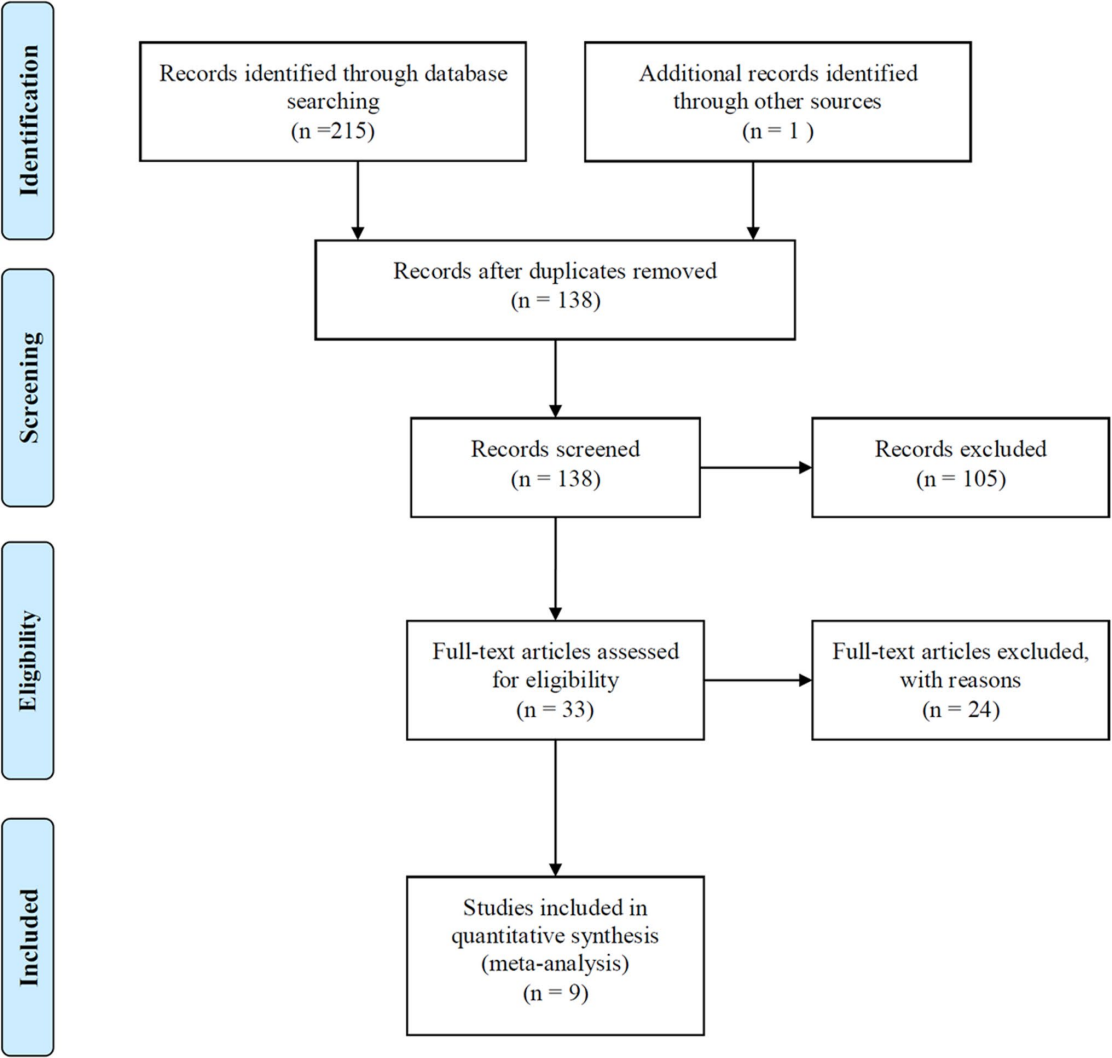
Flow-chart illustrating the article search process

After having assessed the studies by selection criteria, data from 9 studies were included, which involves 514 patients with SLE. A flow chart of the study selection process is shown in Fig. 1.

### Study characteristics

Tables 1 and 2 showed the summary of the included study characteristics. Two took place in Spain [14, 15], and one occurred in each of the following countries: India [25], Canada [26], Thailand [27], Egypt [28], Iran [29], Korea [30], and UK [31]. Two studies had large sample sizes (≥ 100 cases) and the others were small sample size (< 100 cases). When evaluated by M-NOS, out of 5 possible points, one received 4 points, five received 3 points, and three received 2 points.

**Table 1:** Summary of study characteristics

Study	Country	Design	Aim	Diagnostic criteria of SLE	Patient	Sample size (%Female)	Age (years)	Disease Duration (years)	Disease activity	Corticosteroidsuse (%)	Cumulative disease damage
Cervilla O et al. (2020)	Spain	CS	To analyze sleep quality in FM and SLE and explored its relationship with other clinical and psychological manifestations	1982 and 1997 ACR	Outpatient	19 (100%)	40.21 ± 10.25	14.90 ± 7.90	SLEDAI 2.83 ± 3.13	55.55%	NA
Chandrasekhara PK et al. (2009)	India	CS	To analyze sleep complaints in SLE patients and to determine its prevalence and associations	1997 ACR	Outpatient	50(90%)	26 ± 9	2.83 ± 2.42	SLAM-R 4.2 ± 2.7	92%	SLICCDI 0.94 ± 1.2
Costa Da et al. (2005)	Canada	CS	To characterize sleep complaints in women with SLE and to identify correlates of sleep quality	1997 ACR	Outpatient	100 (100%)	45.19 ± 14.12	13.14 ± 9.43	SLAM-R 5.56 ± 3.52	26%	SLICCDI 1.69 ± 1.81
Kasitanon N et al. (2013)	Thailand	CO	To determine the prevalence and associated factors of sleep disturbance in SLE, and the correlation between changes in clinical parameters and sleep quality over time	1997 ACR	Outpatient	56 (100%)	37.49 ± 12.27	8.60 ± 7.28	SLEDAI-2 K 4.23 ± 4.77 PGA 2.25 ± 1.25	100%	SLICCDI 0.25 ± 0.54
Kotb HA et al. (2013)	Egypt	CS	To assess the prevalence of sleep disturbance in female SLE patients and to evaluate the correlation between sleep disturbance and some disease parameters	1982 ACR	Outpatient and inpatient	30 (100%)	26.17 ± 6.75	4.12 ± 2.46	SLEDAI 12.17 ± 5.41	100%	SLICCDI 0.97 ± 0.93
Mirbagher L et al. (2016)	Iran	CS	To determine sleep quality and its associated factors in SLE patients, and to evaluate the effects of sleep quality disturbance on various dimensions of quality of life	1982 and 1997 ACR	Outpatient	77 (100%)	36.5 ± 10.1	8.3 ± 3.8	SLEDAI-2 K 3.1 ± 3.6	77.9%	SLICCDI 0.49 ± 0.88
Moraleda V et al. (2017)	Spain	CS	To analyze sleep quality (subjective and objective) of SLE patients and its possible relationships with the main manifestations of the disease	1982 and 1997 ACR	Outpatient	21 (100%)	38.33(11.39)	13.91(8.23)	NA	85.7%	NA
Moon SJ et al. (2018)	Korea	CS	To identify whether HR-QoL determinants in middle-aged female SLE patients differed according to the presence or absence of FM	1997 ACR	NA	41 (100%)	42.13 ± 10.76	6.49 ± 7.11	SELENA-SLEDAI 6 ± 3.07	100%	SLICCDI 0.35 ± 0.77
Tench CM et al. (2000)	UK	CO	To assess the prevalence and associations of fatigue in SLE	1982 and 1997 ACR	Outpatient	120 (100%)	38.35 ± 9.75	3.57 ± 4.22	ECLAM 3.76 ± 5.25 SLAM 5.65 ± 3.75	51%	SLICCDI 0.35 ± 0.75

Values were number (percentage), mean ± standard deviation, percentage, or median (standard deviation)

*CS*, cross-sectional; *CO*, cohort; *FM*, fibromyalgia; *SLE*, systemic lupus erythematosus; *HR-QoL*, health-related quality of life; *ACR*, American College of Rheumatology; *NA*, not available; *SLEDAI*, Systemic Lupus Erythematosus Disease Activity Index; *SLAM-R*, systemic lupus activity measure revised; *SLEDAI-2 K*, Systemic Lupus Erythematosus Disease Activity Index-2000; *PGA*, Physician’s Global Assessment; *SELENA-SLEDAI*, Safety of Estrogens in Lupus Erythematosus National Assessment version of the Systemic Lupus Erythematosus Disease Activity Index; *ECLAM,* European Consensus Lupus Activity Measure; *SLAM*, systemic lupus activity measure; *SLICCDI*, Systemic Lupus International Collaborating Clinics/American College of Rheumatology Damage Index

**Table 2:** Measurement, cut-points, prevalence, score and r of depression and poor sleep quality and quality of the included studies

	Depression				Poor sleep quality (PSQI)			*r*^a^	Depression Score (number of patients in each group)		Quality **(**M-NOS**)**
Study	Measurement	Cut-point	Prevalence	Score	Cut-point	Prevalence	PSQI score		Good sleep quality (PSQI < 6)	Poor sleep quality (PSQI ≥ 6)	
Cervilla O et al. (2020)	HADS-D	NA	NA	4.28 ± 4.81	≥ 6	NA	8.08 ± 4.00	0.549^*^	NA	NA	2
Chandrasekhara PK et al. (2009)	CES-D	≥ 16	70%	21 ± 11	≥ 6	62%	6.4 ± 3.8	0.675^***^	12.26 ± 9.26 (19)	26.26 ± 8.26 (31)	3
Costa Da et al. (2005)	CES-D	≥ 16	29%	13.02 ± 11.35	≥ 6	56%	6.98 ± 4.03	0.560^***^	NA	NA	3
Kasitanon N et al. (2013)	HAM-D	≥ 7	NA	15.75 ± 11.80	≥ 6	55.36%	7.86 ± 5.42	0.707^***^	9.64 ± 8.74 (25)	20.68 ± 11.75 (31)	2
Kotb HA et al. (2013)	CES-D	≥ 16	73.3%	20.80 ± 6.98	≥ 6	76.7%	8.47 ± 3.53	0.698^***^	12.14 ± 2.27 (7)	23.43 ± 5.64 (23)	3
Mirbagher L et al. (2016)	HADS-D	≥ 8	46.1%	NA	≥ 6	57.1%	7.06 ± 0.46	NA	5.4 ± 3.8 (33)	8.0 ± 4.0 (44)	3
Moraleda V et al. (2017)	HADS-D	NA	NA	5.05(3.68)	NA	NA	8.62(3.13)	0.204	NA	NA	2
Moon SJ et al. (2018)	PHQ-9	≥ 10	46.3%	9.42 ± 7.68	> 5	85.4%	9.06 ± 5.38	0.644^**^	NA	NA	3
Tench CM et al. (2000)	HADS-D	≥ 8	37%	6.00 ± 4.50	≥ 6	59%	8.00 ± 4.50	0.416^***^	NA	NA	4

^*^P < 0.05, ^**^P < 0.01, ^***^P < 0.001. ‡a: Pearson correlation coefficient. †Values were number, percentage, mean ± standard deviation, or median (standard deviation)

*HADS-D*, Hospital Anxiety and Depression Scale-Depression; *CES-D*, Center for Epidemiological Studies Depression scale; *HAM-D*, Hamilton rating scale for depression; *PHQ-9*, the nine-item Patient Health Questionnaire; *NA*, not available; *PSQI*, Pittsburgh Sleep Quality Index; *M-NOS*, Modified Newcastle–Ottawa Scale

### Correlation between depression and sleep quality in SLE patients

**Fig. 2:**
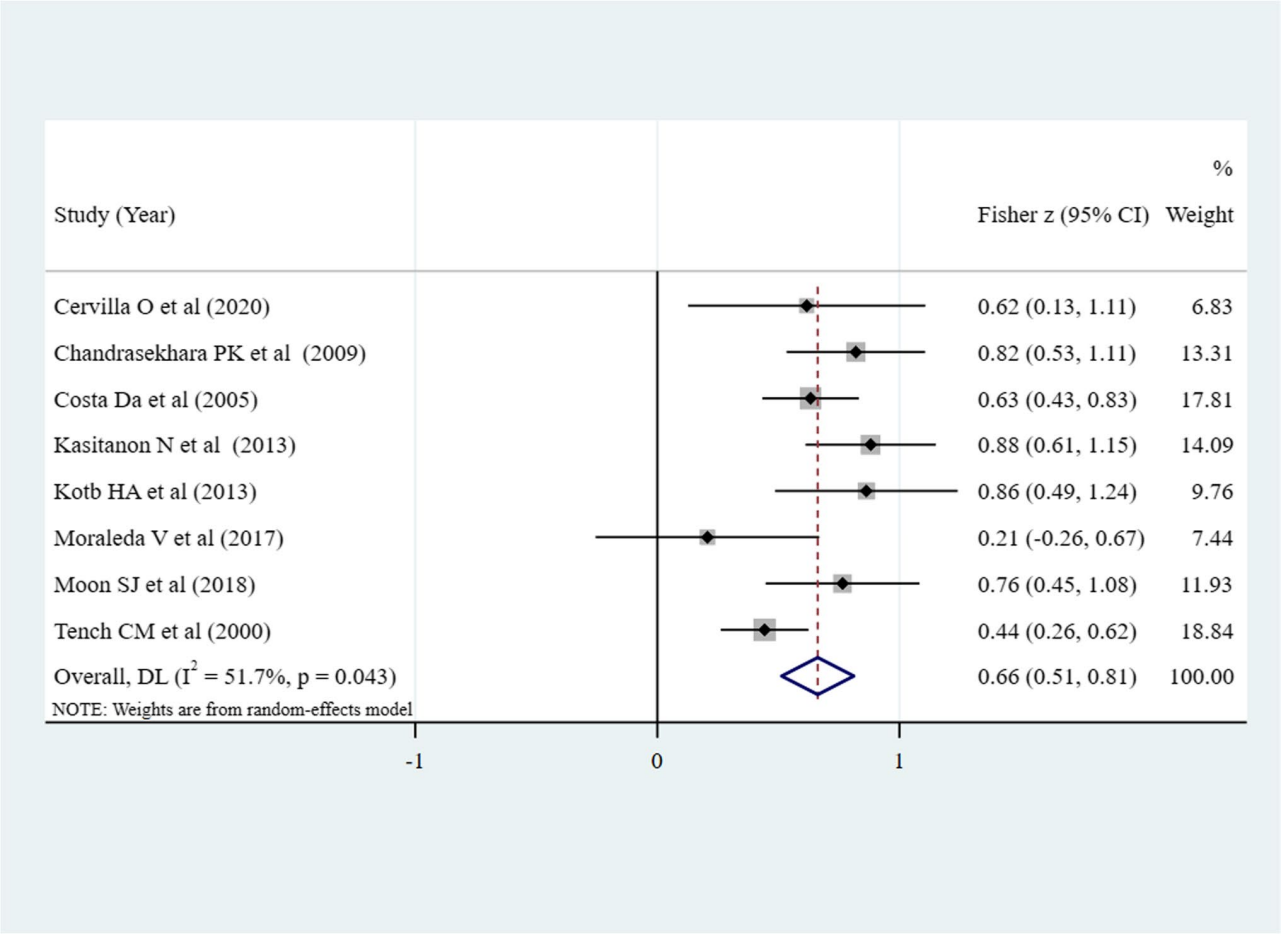
Meta-analysis of the correlation between depression and sleep quality (*N* = 8)

**Fig. 3:**
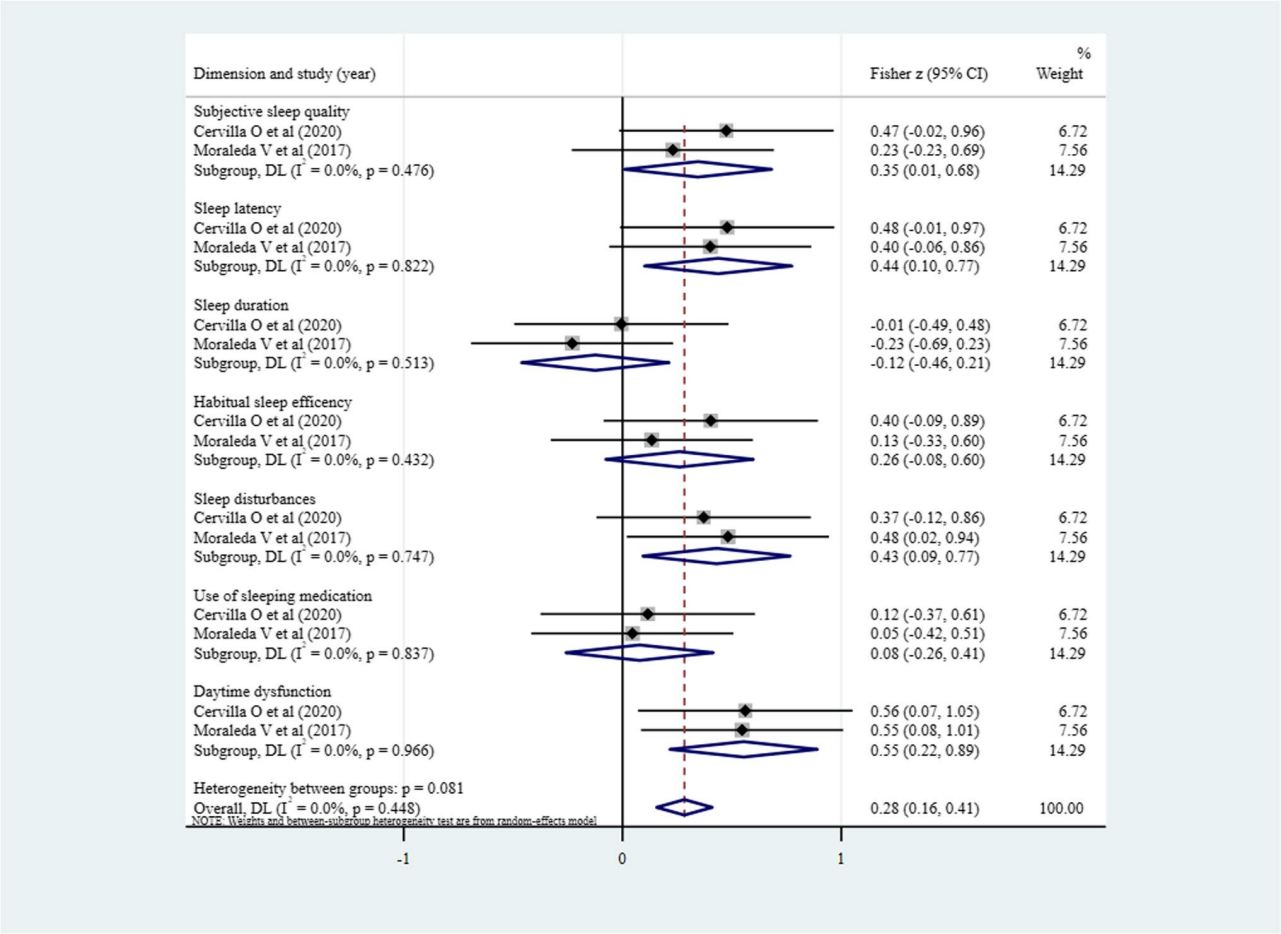
Meta-analysis of the correlation between depression and 7 dimensions of PSQI (*N* = 2)

As shown in Fig. 2, eight studies [14, 15, 25–28, 30, 31] reported *r* between depression and sleep quality, the pooled *z* was 0.66 (95% CI 0.51–0.81, *P* = 0.043; *I*^2^ = 51.7%). The correlation magnitude is moderate (pooled *r* = 0.580) and significant, which is reflected by the 95% CI (0.473–0.670) that does not include the value 0, suggesting a positive relationship. Among the seven dimensions of PSQI, depression was associated with subjective sleep quality (pooled *r* = 0.332, 95% CI 0.009–0.592, *P* = 0.044; *I*^2^ = 0%), sleep latency (pooled *r* = 0.412, 95% CI 0.101–0.649, *P* = 0.011; *I*^2^ = 0%), sleep disturbances (pooled *r* = 0.405, 95% CI 0.094–0.645, *P* = 0.012; *I*^2^ = 0%), and daytime dysfunction (pooled *r* = 0.503, 95% CI 0.214–0.711, *P* = 0.001; *I*^2^ = 0%), no significant correlation was found with the other three dimensions-sleep duration, habitual sleep efficiency, and use of sleeping medication (Fig. 3).

### Comparison of depression levels in patients with SLE and good and poor sleep quality

**Fig. 4:**
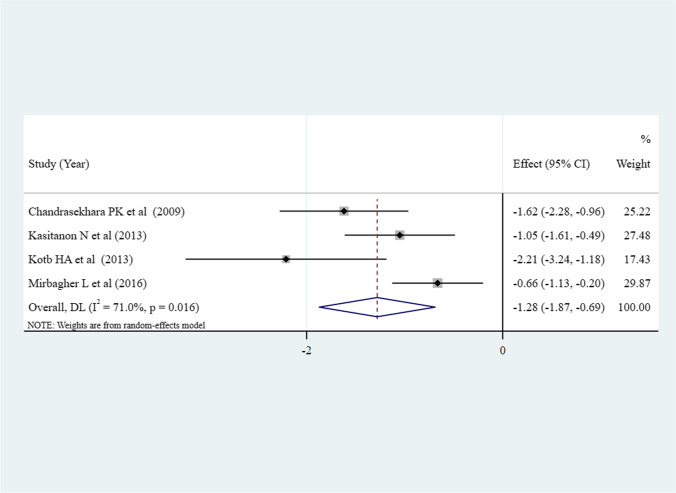
Meta-analysis comparing the depression levels in patients with SLE and good sleep quality and patients with SLE and poor sleep quality (*N* = 4)

There were 4 studies that compared depression between good (total sample size = 84) and poor (total sample size = 129) sleep quality in patients with SLE [25, 27–29]. Compared to good sleepers, patients with SLE and poor sleep quality had higher levels of depression (pooled SMD =  − 1.28, 95% CI [− 1.87, − 0.69], *P* < 0.001; *I*^2^ = 71%) (Fig. 4).

### Subgroup analysis

Subgroup analysis was conducted based on corticosteroids use, measurement of depression, diagnostic criteria of SLE, region, and quality. Table 3 indicated that corticosteroids use, measurement of depression, diagnostic criteria of SLE, and region might be the source of heterogeneity in meta-analysis of the correlation between the depression and sleep quality in SLE. As shown in Table 4, depression measurement, SLE diagnostic criteria, and study’s quality may be a source of heterogeneity in meta-analysis comparing the depression level in SLE patients with good and poor sleep quality, but not including corticosteroids use and region.

**Table 3:** Subgroup analysis of the pooled results of the correlation between depression and sleep quality in SLE patients

Variables	No. of studies	No. of patients	Fisher *z* (95% CI)	*P* value	Heterogeneity	
					*I*^2^ (%)	*P* value
Corticosteroids use						
< 100%	5	310	0.57(0.39, 0.74)	< 0.001	47.3	0.108
100%	3	127	0.84(0.66, 1.02)	< 0.001	0	0.853
Measurement of depression						
HADS-D	3	160	0.43(0.27, 0.59)	< 0.001	0	0.479
CES-D	3	180	0.72(0.57, 0.87)	< 0.001	0	0.415
HAM-D	1	56	0.88(0.61, 1.15)	< 0.001	0	-
PHQ-9	1	41	0.76(0.45, 1.08)	< 0.001	0	-
Diagnostic criteria of SLE						
1982 and 1997 ACR	3	160	0.43(0.27, 0.59)	< 0.001	0	0.479
1997 ACR	4	247	0.75(0.62, 0.88)	< 0.001	0	0.479
1982 ACR	1	30	0.86(0.49, 1.24)	< 0.001	0	-
Region						
Europe	3	160	0.43(0.27, 0.59)	< 0.001	0	0.479
Asia	3	147	0.83(0.66, 1.00)	< 0.001	0	0.859
North America	1	100	0.63(0.43, 0.83)	< 0.001	0	-
Africa	1	30	0.86(0.49, 1.24)	< 0.001	0	-
Qualit**y**						
High risk	4	236	0.67(0.43, 0.90)	< 0.001	62.9	0.044
Low risk	4	201	0.66(0.44, 0.89)	< 0.001	50.5	0.109

*HADS-D*, Hospital Anxiety and Depression Scale-Depression; *CES-D*, Center for Epidemiological Studies Depression Scale; *HAM-D*, Hamilton Rating Scale for Depression; *PHQ-9*, the nine-item Patient Health Questionnaire; *ACR*, American College of Rheumatology; *CI*, confidence interval

**Table 4:** Subgroup analysis of the pooled results in depression levels between SLE patients with good and poor sleep quality

Variables	No. of studies	Pooled SMD (95% CI)	*P* value	Heterogeneity	
				*I*^2^ (%)	*P* value
Corticosteroids use					
< 100%	2	− 0.11 (− 2.05, − 0.18)	0.020	81.6	0.020
100%	2	− 1.55 (− 2.67, − 0.42)	0.007	73.6	0.052
Measurement of depression					
CES-D	2	− 1.79 − 2.34, − 1.24)	< 0.001	0	0.341
HAM-D	1	− 1.05 (− 1.61, − 0.49)	< 0.001	0	-
HADS-D	1	− 0.66 (− 1.13, − 0.20)	0.005	0	-
Diagnostic criteria of SLE					
1982 and 1997 ACR	1	− 0.66 (− 1.13, − 0.20)	0.005	0	-
1997 ACR	2	− 1.31 (− 1.86, − 0.75)	< 0.001	39.9	0.197
1982 ACR	1	− 2.21 (− 3.24, − 1.18)	< 0.001	0	-
Region					
Asia	3	− 1.07 (− 1.60, − 0.54)	< 0.001	63.4	0.065
Africa	1	− 2.21 (− 3.24, − 1.18)	< 0.001	0	-
Quality					
High risk	2	− 0.82 (− 1.19, − 0.45)	< 0.001	6.9	0.300
Low risk	2	− 1.79 (− 2.34, − 1.24)	< 0.001	0	0.341

*CES-D*, Center for Epidemiological Studies Depression Scale; *HAM-D*, Hamilton Rating Scale for Depression; *HADS-D*, Hospital Anxiety and Depression Scale-Depression; *ACR*, American College of Rheumatology; *SMD*, standardized mean difference; *CI*, confidence interval

### Sensitivity analyses and publication bias

Sensitivity analyses displayed that, for all comparisons between patients with good and poor sleep quality, as well as the pooled results of differences between depression level and sleep quality in patients with SLE, the omission of any study made no significant difference, indicating the stability of our meta-analysis (figures not shown). As the number of studies included in this meta-analysis was 9, less than 10, funnel plots and the Egger’s test were not conducted to assess publication bias.

## Discussion

This systematic review and meta-analysis of 9 studies involving 514 patients with SLE is the first to explore the relationship between depression and sleep quality.

In this meta-analysis, a moderate correlation of sleep quality with depression was found (pooled *r* = 0.580). Compared to good sleepers, patients with SLE and poor sleep quality had higher levels of depression, corroborated by past studies [25, 27–29]. This study also found that there was correlation between depression and subjective sleep quality, sleep latency, sleep disturbances, and daytime dysfunction, four dimensions of the PSQI. There were no significant correlations with the other three dimensions-sleep duration, habitual sleep efficiency, and use of sleeping medication. The underlying mechanisms of sleep disorders in patients with SLE are yet unclear [4]. Previous studies have focused on possible neural mechanisms and found that sleep deprivation influences brain systems that are involved in emotion, such as the amygdala [32], and the anterior cingulate cortex are thought to be a region where genes that control circadian rhythms are dysregulated in depression [33] and where neurons increase their activity during sleep and disengagement from tasks [34]. Cheng et al. [35] found that both poor sleep quality and depressive problems are significantly positively correlated with functional connectivities involving the lateral orbitofrontal cortex, the dorsolateral prefrontal cortex, the cingulate cortex, and the precuneus; and the functional connectivity links between the brain areas identified played a significant role in the association of depressive problems with poor sleep quality, while the associations of sleep quality with depressive problems mediated by these links were less significant. These findings provides a neural basis for understanding how depression is associated with poor sleep quality, and this in turn has implications for treatment because of the brain areas identified.

Subgroup analysis demonstrated a positive correlation between depression and sleep quality in SLE patients with regard to corticosteroids use, measurement of depression, diagnostic criteria of SLE, and region; and the four variables might be the source of heterogeneity. Compared with less than 100% use of corticosteroids, SLE patients with 100% corticosteroids use had a stronger relationship between depression and sleep quality. Previous studies reported that corticosteroids can contribute to sleep difficulties and some psychiatric symptoms [26, 28]. It could be the side effects of corticosteroids, such as night sweating which affects or aggravates SLE patients’ sleep disorders, and centripetal obesity that leads to body image concerns. The strength of the correlation between depression and sleep quality varied among different measurement tools of depression, with HAM-D being the high relationship and HADS-D being the low correlation, which was consistent with the explanation of Mirbagher et al. [29] that using different psychological measurement tools may contribute to the differences between the results. To date, subjective measurement of depression in patients with SLE has not been unified. We recommend that future investigators conduct rigorous randomized controlled trials in SLE patients to unify depression assessment tool to make the results more comparable across studies. An interesting finding was that the correlation between depression and sleep quality was strongest when SLE patients met the diagnostic criteria of 1982 ACR, followed by 1997 ACR, and weakest when patients with SLE matched both 1982 and 1997 ACR. As is well known, 1997 ACR revised the criterion number 10 of 1982 ACR—“Immunologic disorder” by deleting item 10(a) and changing item 10(d) [36], and the sensitivity of revised “Immunologic disorder” remained the same, but the specificity decreased [37]. That is, the rate of misdiagnosis of immune abnormalities in 1982 ACR was low, which may lead to patients with immune abnormalities getting closer to a diagnosis of SLE, increasing their psychological burden and affecting their sleep quality. As the diagnostic criteria of SLE will be updated in real time with the progress of detection technology and other reasons, we suggest that researchers adopt the latest diagnostic criteria. There is another discovery that depression had a highest relationship with sleep quality in SLE patients in Africa, and lowest correlation in Europe. It may be because of the poor economic conditions and backward medical conditions in Africa that SLE patients may not get timely and effective treatment, leading to the deterioration of the disease, increased psychological burden, and restless sleep at night. In contrast, Europe has developed economy, advanced medical level and high medical insurance coverage, so the treatment of SLE patients can be carried out in an orderly manner. This review showed that literature’s quality did not affect the association between depression and sleep quality in patients with SLE and might not be the source of heterogeneity of the pooled correlation coefficient. However, given that the higher the quality of the article, the more reliable the conclusions, we still hope researchers in future studies could write the manuscript according to the highest quality standards as possible.

Since the relationship between depression and sleep quality in SLE patients is considered to be clearer in this meta-analysis, the development of strategies to improve sleep quality in patients with SLE underscores the continuing importance of mental health assessments. Previous meta-analysis has reported the safety of therapeutic exercise programs, and exercise do not adversely affect disease activity, accompany with an interesting finding that exercise-based interventions positively influence depression [38]. Therefore, SLE patients may be able to decrease their depression levels through exercise, and thus improve their sleep quality. Lately, sleep hygiene education has shown its own potential as an intervention strategy to cover the growing public health concerns of an increasing general population with sleep complaints [39]. Nevertheless, a lack of repeated studies on the use and relevance of sleep hygiene ingredients is still existed.

However, there are several limitations in this review. First, the data were derived from studies using different designs involving different patient groups (e.g., from different countries), which may lead to heterogeneity between studies. However, we conducted subgroup analysis and sensitivity analysis to find the source of heterogeneity, and we did find some possible sources. Second, only the studies that explored sleep quality by PSQI were included, as PSQI being the most commonly used to assess SLE patient’s sleep quality. Third, the assessment tools of sleep quality and depression included in this review were based on subjective feedback of the patients, which may not be objective enough. Fourth, the included researches in this meta-analysis were mainly cross-sectional designs, the number of included articles and total sample size were relatively small. Therefore, more high-quality prospective studies with large sample sizes are needed.

## Conclusion

This meta-analysis suggests that depression has a moderate correlation with sleep quality in patients with SLE. Patients with poor sleep quality tended to have higher levels of depression than those of good sleepers. Awareness of this correlation may prompt rheumatology physicians and nurses to assess and treat depression and improve patients’ sleep quality.
